# A Systematic Review Comparing the Pre-event Behaviours of Suicide by Mass Violence With Individuals Who Commit Suicide Without Mass Violence

**DOI:** 10.1177/00302228251323641

**Published:** 2025-02-20

**Authors:** Thomas James Vaughan Williams

**Affiliations:** 1 4013 University of Huddersfield, Queensgate, Huddersfield, UK

**Keywords:** suicide, mass violence, ideation, tendencies, systematic review

## Abstract

**Intro:** Perpetrators of mass violence and individuals who commit suicide have been discussed in terms of the experiencing similar ideations and tendencies. This has led to the promotion of utilising suicide prevention strategies to aid the reduction of mass violence probability. However, there is minimal research in terms of the identifying differentiation behaviours between potential mass violence and individuals who potentially will commit suicide without mass violence. **Method:** The present study involves a comparative systematic review of both suicide research and mass violence research, to explore and identify behavioural similarities and differences in pre-event behaviour between the two phenomena. **Results:** The results showcase key similarities but more notably, six key behavioural differences, especially in terms of externalisation and internalisation. **Conclusion:** There are potentially six key behavioural pre-event differences between mass violence and individuals who commit suicide without mass violence. Implications and future research are discussed.

## Introduction

It is a prevalent feature of mass violence for the perpetrator to finish their attack with an attempt of suicide (Silver & Silva, 2022). Research has highlighted not only the prevalence of this phenomena (between 2000 to 2019, 40.5% of mass shootings attempted suicide after their attack) but how this phenomenon is increasing in prevalence also (Girgis et al., 2024).

Both the phenomena of mass violence and suicide are extensively linked within previous literature exploring these behaviours through various psychological lenses. He et al. (2003) discussed the factors that may influence individuals to commit these types of phenomena, highlighting issues such as economic hardship and social isolation as potential circumstances that influence these behaviours. It is theorised that these circumstances negatively contribute to an individual’s mental health, causing issues such as thought distortions, suicidal ideation, and emotional dysregulation, all of which are potential precursors to both mass violence and suicide (Lankford et al., 2021). This, in turn, then contributes to the overall heightened risks of both self-directed and outward-directed violence (Lanier, 2010). However, the individual’s perception of the cause of these issues may also influence which behaviour they perform, with individuals who blame themselves and have a perceived sense of personal failure are more likely to engage with an act of suicide, while those who attribute their issues to external forces may resort to homicide or mass violence (Henry & Short, 1964). Yet, despite these potential differences in perception, majority of research in the area aligns with the notion that both mass violence and suicide appear to be a manifestation of extreme self-destructive tendencies and suicidal ideation influenced by various factors (Silver, 2024).

This has led the theorisation that for a large proportion of mass violence behaviours, such as a publication of their manifesto (Williams et al., 2023), weapon acquisition (Williams et al., 2024) and subsequent attack, are all elements of their overall act of suicide (Densley & Peterson, 2022). This has encouraged researchers and mass violence prevention practitioners to promote the notion that suicide prevention strategies are a valuable tool to preventing mass violence (Densley & Peterson, 2022). By identifying and addressing an individual’s suicidal tendencies and behaviour, we reduce the risk of that individual committing an act of violent suicide in the form of a mass violence (Peterson, 2022).

Research in this area supports the notion of preventing the probability of mass violence occurring by decreasing suicidal tendencies in vulnerable individuals and providing appropriate venues such as schools with crisis and suicide intervention resources and strategies (Gerard et al., 2015). As a strategy against mass violence, suicide prevention strategies are incredibly valuable to utilise (Peterson, 2022). However, in terms of differentiating between individuals who are at risk of suicide without mass violence and suicide by mass violence, there is very limited research in terms of defining key differences in observable behaviours and signposts between the two outcomes.

From the limited pool of research in the field, research has highlighted minimal but distinct psychological and demographic differences between the two phenomena, particularly in terms of gender differences (Males have higher likelihood than females to commit both suicide and violence: Batton, 2004) mental health histories (Fridel, 2022), and behaviour premeditation (Lankford et al., 2021). Yet, research has predominantly highlighted the vast similarities between the two, ranging from individualised causes such as social rejection (Wu, 2004) to wider scoped causations such as historical cultural trauma and societal values (Lanier, 2010).

Although both suicide without mass violence and suicide by mass violence follow similar trajectories according to the literature, identifying key differences in the pre-event behaviour will not only potentially aid prevention strategies for both phenomena but also allow more specific resources and strategies to be applied to the relevant individuals. The present systematic review aims to provide this information, highlighting not only the similarities in pre event behaviour between singular and mass suicide but also the differences in pre-event behaviour.

## Methodology

### Search Strategy

The articles screened and utilised for this systematic review were sourced from numerous academic search engines (e.g. SpringerLink), with broader search engines such as google scholar also being utilised. Keywords were utilised to identify appropriate papers for this review, some of the keywords being: Suicide, Suicidal behaviours, Pre-event, Suicidal ideation, Mass violence.

### Inclusion and Exclusion Criteria

To ensure only relevant studies were included within the current review, abstracts and results were screened for information relating to the either pre-event behaviours of individuals who committed suicide without mass violence or a mass violence. The inclusion and exclusion criteria are presented as such below:

### Inclusion Criteria

(1) Research that explored the behaviours of mass violence perpetrators or individuals who committed suicide without committing mass violence, focusing on the behaviours prior to the mass violence or the act of suicide.
(2) All research had to be sourced from a peer reviewed academic source
(3) All research written in the English language

### Exclusion Criteria

(1) Research that focused on the behaviours of mass violence or individuals who committed suicide without committing mass violence, during or after the event
(2) Research that discussed the behaviours of mass violence or individuals who committed suicide without committing mass violence, but did not highlight and discuss pre-event behaviours specifically.
(3) Research that discussed the pre-event behaviours of mass violence or individuals who committed suicide without committing mass violence, but did not provide enough depth to allow direct comparison with other research findings.

The initial search resulted in literature pool of 74 independent research articles, which after screening for the explored phenomena and utilising the inclusion criteria, 17 were appropriate for this review. Of the selected 17, nine explored the pre-event behaviours of mass violence perpetrators and eight explored the pre-event behaviours of suicide. All included sources can be found in the appendix Table A1, A2.

## Results

### Behavioural Similarities

As showcased in table one, there were eight prominent features across both the suicide only pre-event behaviours and the mass violence pre-event behaviours. Amongst the eight prominent features there are three key themes: Withdrawal, Social Isolation and Mental Health Challenges.

Yet, as discussed within Table 1, the purposes and effects of these are different. In the suicide research, these three themes were often signs of self-directed despair and self-depreciation, where in contrast to mass violence, these three themes were often motivations for fuelling further resentment and grievance towards others. Although the eight features and three themes are all shared, the directional effect is different.

**Table 1.** Summary of Behavioural Similarities.

Behaviour	Suicide research	Mass violence research
Isolation and social withdrawal	Tendency to withdraw from friends, family, and social activities	Often isolated, lacks close social connections, or purposely distances from others as part of growing grievances
History of mental health issues	Commonly involves depression, anxiety, mood disorders, or prior suicide attempts as indicators of risk	High prevalence of mental health challenges, particularly depression, personality disorders, and untreated trauma
Sense of hopelessness or despair	Feelings of hopelessness, worthlessness, and an inability to envision a positive future	Perceived lack of future opportunities; views violence as a “solution” to perceived injustices or grievances
Resentment and frustration	Frustration with life circumstances, often directed inward, leading to self-blame and self-destructive thoughts	Frustration directed outward, often blaming society, specific groups, or individuals, which can fuel violent intent
History of aggressive or risky behaviour	Suicidal individuals may engage in risky behaviours (e.g., substance abuse, self-harm) as coping mechanisms	Mass violence perpetrators may have histories of violent behaviour or increasingly aggressive expressions of anger and resentment
Perceived victimisation	May feel victimised by life circumstances, leading to self-directed harm due to feelings of personal failure	Feels unfairly treated or oppressed by others, leading to a desire for revenge or retribution through violent acts
Suicidal ideation or death wishes	Expresses thoughts of suicide or a wish to escape suffering, either directly or indirectly	Suicidal ideation often overlaps with violent intent, seeing the attack as a final act with likely self-sacrifice (suicide or death by law enforcement)
Difficulty coping with stress	Struggles with handling stress and significant life events, which may trigger worsening mental health symptoms	Poor coping mechanisms lead to resentment and planning for violent acts as a perceived way to manage or end suffering

These findings showcase the psychological and behavioural parallels between the pre-event behaviours of individuals who committed suicide and those who commit mass violence, particularly in their struggles with Withdrawal, Social Isolation and Mental Health Challenges. Both groups frequently exhibit social withdrawal, but the motivations behind their disengagement differ. Suicidal individuals often retreat due to overwhelming emotional distress, feeling disconnected from support systems, whereas mass violence perpetrators may isolate themselves as their grievances intensify, fostering a growing sense of resentment and alienation.

A history of mental health issues, including depression, anxiety, personality disorders, and past trauma, is a common factor in both cases, though how these struggles manifest varies. Individuals at risk of suicide typically direct their pain inward, blaming themselves for their perceived failures or misfortunes. In contrast, mass violence perpetrators tend to externalise their suffering, channelling frustration outward and blaming specific individuals, institutions, or society at large. Feelings of hopelessness and resentment are particularly influential in shaping their actions, with individuals at risk of suicide often experiencing self-directed despair, believing that they have no future or way to escape their suffering, whereas mass violence perpetrators view violence as a means of retribution.

### Behavioural Differences

As showcased in table two, there were six behavioural differences between mass violence and individuals who committed suicide only. The research focusing on suicide showcases how the pre-event behaviour of suicide tends to be more internalised, reflecting the individual’s emotional distress and directing all the negative effects of their circumstances inward towards themselves.

In contrast, mass violence pre-event behaviour is often focused on blaming and resenting others, directing negative effects of their circumstances outwards. A key difference is within the communication and planning of the eventual event, with mass violence preparators often leaking their intentions, declaring their grievances and planning the event for long periods of time. In contrast, individuals who commit suicide tend to more subtle about their intentions and spend less time planning their event. Table 2

**Table 2.** Summary of Behavioural Differences.

Behaviour	Suicide research	Mass violence research
Direction of behaviour	Internalised (self-directed)	Externalised (other-directed)
Emotional indicators	Depression, hopelessness, withdrawal	Anger, frustration, perceived victimisation
Communication	Often subtle, may hint at self-harm intentions	Explicit declarations, manifestos, or threats
Planning of event	Less methodical; often impulsive	Highly planned, strategic, involving weapon acquisition
Suicide method intention	Typically seeks privacy, avoids public focus	Often seeks media attention or notoriety
Mental health indicators	Depression, anxiety, substance abuse	Perceived grievances, resentment, desire for revenge

These findings highlight key psychological and behavioural differences between individuals at risk of suicide and potential perpetrators of mass violence, particularly in the direction of behaviour, emotional triggers, and planning. Individuals at risk of suicide without mass violence internalise their distress, leading to self-harm, whereas mass violence perpetrators externalise their frustration, directing harm toward others. Emotionally, suicidal individuals often experience depression, hopelessness, and withdrawal, while mass violence offenders exhibit anger, frustration, and a strong sense of perceived victimisation. Their communication patterns also differ, with individuals at risk of suicide opting to hint subtly at their intentions, whereas mass violence perpetrators often make explicit threats, declarations, or manifestos. Another prominent contrast is in event planning, where suicide attempts are often impulsive and private, whereas mass attacks are highly methodical and designed for maximum impact, often seeking media attention. In regard to mental health, while suicidal individuals struggle with depression, anxiety, and substance abuse, perpetrators of mass violence predominantly experience deep-seated resentment and a desire for revenge.

### Discussion

The present study showcases the behavioural similarities and differences between individuals who commit mass violence and individuals who commit suicide without mass violence. The key findings within the presented results are the behavioural differences that potentially allow differentiation between individuals who go on to commit mass violence compared to those who commit suicide without mass violence, most notably the directional difference in behaviour.

The results showcase that for mass violence, their emotions, communication and mental health indicators are all externalised towards perceived grievances and entities that the individuals deem responsible for these grievances. In contrast, individuals who go on to commit suicide only, internalise all their behaviours, withdraw emotionally and direct all harmful behaviours towards themselves, such as in forms of substance abuse and self-harm.

### Mental Health Indicators

A prominent feature in both the similarities and differences between perpetrators of mass violence and individuals who commit suicide was the presence of mental health difficulties. Both outcomes (committing mass violence and committing suicide) were found to be preceded by a history of mental health issues, with individuals at risk commonly experiencing depression, anxiety, mood disorders, or prior suicide attempts. These conditions serve as strong indicators of vulnerability, suggesting a similar level of psychological distress between the two phenomena. Depression plays a prominent role in both outcomes, as it often manifests through persistent sadness, hopelessness, and emotional pain, which can lead to self-destructive behaviour (Yoshimasu et al., 2008) or violent impulses directed outward (Lankford & Silva, 2021). Anxiety and mood disorders, which can affect emotional regulation, also contribute to emotional dysregulation and feelings of isolation, increasing the risk for both self-harm and harm to others (Portzky et al., 2005). Additionally, issues such as untreated trauma, which are present in both groups, potentially exacerbate emotional instability and impulsivity, which further heightens the risk of either outcome (O’Connor & Nock, 2014).

However, there are key differences in the mental health indicators that tend to distinguish mass homicide from suicide. Individuals at risk of suicide are more likely to struggle with pervasive feelings of despair, hopelessness, and emotional exhaustion, potentially accompanied by substance abuse as a coping mechanism. Whereas those at risk of engaging in mass violence typically experience heightened feelings of grievance, anger and resentment. While depression and trauma may still be present, the profile for a perpetrator of mass violence showcased in the results highlights these individual’s focused desire to enact harm, which differentiates their behaviour from the internalised pain exhibited from individuals at risk of suicide. It is this differentiation between the externalising and internalising of grievances and behaviours that is the fundamental finding of the present research.

### Future Research

These results produce two main implications for further research: The need for exploration into what determines externalisation or internalisation and the potential for a scale to be created.

### Need for Exploration into what Determines Externalisation Vs Internalisation

The presented results showcase a focus on the differences between the two explored events (mass violence and suicide without mass violence), highlighting a key difference in the direction the pre-event behaviours (externalised or internalised). Yet, why some individuals chose to internalise their grievances and suicidal tendencies and others externalise them, is still contested and debated.

One potential explanation for the differentiation between internalising and externalising behaviours in individuals experiencing suicidal tendencies is provided by Agnew’s (1992) General Strain Theory (GST). This theory suggests that individuals who endure intense hardships, such as failure and/or social rejection, may respond to these stressors in different ways. Depending on their personal coping mechanisms, levels of social support, and inherent personality traits, some may direct their distress outward through aggression and violence, while others internalise it, leading to self-harm or acts of suicide. Similarly, Baumeister’s (1990) Escape Theory of Suicide supports this perspective, particularly in relation to internalisation, claiming that individuals overwhelmed by chronic failure or hopelessness may seek to escape from their distress through self-destructive behaviours, including suicide. However, it also accounts for externalisation, suggesting that those who exhibit traits such as narcissism, impulsivity, or an external locus of control may be more prone to violent or aggressive responses rather than self-harm (Howard, 2015).

Yet, as highlighted in the present study, a lot of the strains highlighted in GST theory and Escape theory overlap prominently in both pre-event behaviours of individuals who have committed suicide and those who commit suicide via mass violence. This furthers the call to action to explore in more specific depth the potential differential factors that influence if an individual with suicidal ideations externalises or internalises their grievances. By uncovering what individual factors facilitate an individual with suicidal tendencies to either internalise or externalise their behaviours we can understand further the risk factors involved with both explored phenomena. This would potentially allow a valuable enhancement to mental health practices to develop more targeted intervention strategies, improve risk assessments, and provide personalised support to those struggling with suicidal ideation.

### The Potential for a Scale to be Created

The results of the present review also initiate the potential for a scale to be created, that measures for the key behavioural differences showcased in the results and allowing for detection of if an individual is more or less likely to not only have suicidal tendencies but externalise or internalise their tendencies. If an effective, ease of use scale was to be created and validated efficiently, it would allow the administration of this scale to be utilised by relevant practitioners and professionals to determine the risk relevancy of individuals, allowing more effective utilisation of crisis and suicide resources and strategies.

### Limitations

The main limitation that arises from the utilisation of a systematic review is the interpretation of the findings, especially in terms of creating overall conclusions about the explored phenomena from a coalition of different research methods and findings (Garg et al., 2008). To minimise this potential effect on the produced inferences, the current study utilised a strict inclusion/exclusion criterion, screening for works that only specifically explored pre-event behaviours of either mass violence or individuals who committed suicide without mass violence. Although this resulted in a smaller research pool to generate inferences, the research included are not only directly relevant to the explored phenomena allowing comparison between papers to be more valid, but the works involved are also some of the most superlative in their respective field.

### Conclusion

In conclusion, the present study utilised the combination of previous works to investigate and present the similarities and differences in pre-event behaviour of individuals who commit suicide and mass violence. The present study not only highlights key behavioural findings, such as six key differences between the two phenomena but suggests the need for further exploration into the individual factors that might cause these key differences and the creation of a scale to measure these differences to aid crisis strategy and resource allocation.

## Appendix

**Table A1.** Description of the selected studies for review (Mass violence research sample *N* = 9)

Authors	Method	Sample	Findings
Capellan, J. A., & Gomez, S. P. (2017). Change and stability in offender, behaviours, and incident-level characteristics of mass public violence in the United States, 1984–2015. Journal of investigative Psychology and Offender Profiling, 15 (1), 51–72. https://doi.org/10.1002/jip.1491 (Capellan & Gomez, 2017)	Open source database	294 mass violence	• Researching location • Catalyst event occurring • Mental illness • Substance abuse • Single or divorced • High school degree or less Access to firearms
Silver, J., & Silva, J. R. (2022). A sequence analysis of the behaviors and experiences of the deadliest public Mass violence. Journal of Interpersonal Violence , 37 (23–24), https://doi.org/10.1177/08862605221078818 (Silver & Silva, 2022)	Case studies	14 mass violence	• Work/School problem • Family problems • Aggressive behaviour • Research • Leakage Interest in past killings
Gerard, F. J., Whitfield, K. C., Porter, L. E., & Browne, K. D. (2015). Offender and offence characteristics of school violence incidents. Journal of Investigative Psychology and Offender Profiling, 13 (1), 22–38. https://doi.org/10.1002/jip.1439 (Gerard et al., 2015)	Case studies	28 Mass violence	• Victimised • Rejected • Loner • Depressio Careful planning
Lankford, A., & Silva, J. R. (2021). The timing of opportunities to prevent mass violence: a Study of mental health contacts, work and school problems, and firearms acquisition. International Review of Psychiatry, 33 (7), 638–652. https://doi.org/10.1080/09540261.2021.1932440 (Lankford, 2018)	Case studies	14 mass violence	• Work or school problem • Purchased firearm • One school issue before buying weapon One mental health contact before buying weapon
Fernandez, E., Callen, A., Johnson, S. L., Gaspar, C., Kulhanek, C., & Jose-Bueno, C. (2020). Prevalence, elicitors, and expression of anger in 21st century mass violence. Aggression and Violent Behavior, 55 (1), 1–7. 10.1016/j.avb.2020.101483 (Fernandez et al., 2020)	Case studies	132 mass violence	• Anger from abandonment and rejection • Psychological > physical provocation Anger externalised
McPhedran, S. (2017). Australian Mass violence: An analysis of incidents and offenders. Journal of Interpersonal Violence, 35 (19–20), 3939–3962. https://doi.org/https://doi.org/10.1177/0886260517713226 (McPhedran, 2017)	Case studies	14 mass violence	• Experienced life stressors up to the event
Lankford , A. (2018). Identifying potential Mass violence and suicide terrorists with warning signs of suicide, perceived victimisation, and desires for attention or fame. Journal of Personality Assessment, 100 (5), 471–482. https://doi.org/10.1080/00223891.2018.1436063	Review	Evidence	• Leakage • Situational difficulties • Identifying with others who have committed mass violence • Legacy tokens Attention seeking behaviour
Allely, C. S., & Faccini, L. (2019). Clinical profile, risk, and critical factors and the application of the “path toward intended violence” model in the case of mass violence dylann roof. Deviant behavior, 40 (6), 672–689. https://doi.org/10.1080/01639625.2018.1437653 (Allely & Faccini, 2019)	Case study	Dylan roofe	• Communication issues • Fixated interests • Situational difficulties/spiralling • Harbouring grievance
Ioannou, M., Hammond, L., & Simpson, O. (2015). A model for differentiating school violence characteristics. Journal of criminal psychology, 5 (3), https://www.emerald.com/insight/content/doi/10.1108 (Ioannou et al., 2015)	Content analysis	40 cases	Sense of the world is unsafe Bullied

**Table A2.** Description of the selected studies for review (Suicide research sample *N* = 8).

Authors	Method	Sample	Findings
O'Connor, R. C., & Nock, M. K. (2014). The psychology of suicidal behaviour. The Lancet Psychiatry, 1 (1), 73–85. https://doi.org/10.1016/S2215-0366(14)70222-6 (O’Connor & Nock, 2014)	Literature review	N/A	Personality and individual differences • Hopelessness • Impulsivity • Perfectionism • Neuroticism and extroversion • Optimism • Resilience Cognitive factors • Cognitive rigidity • Rumination • Thought suppression • Autobiographical memory biases • Belongingness and burdensomeness • Fearlessness about injury and death • Pain insensitivity • Problem solving and coping • Agitation • Implicit associations • Attentional biases • Future thinking • Goal adjustment • Reasons for living • Defeat and entrapment Social factors • Social transmission • Modelling • Contagion • Assortative homophily • Exposure to deaths by suicide of others • Social isolation Negative life events • Childhood adversities • Traumatic life events during adulthood • Physical illness • Other interpersonal stressors Psychophysiological stress response
Riera-Serra, P., Navarra-Ventura, G., & Castro, A. (2024). Clinical predictors of suicidal ideation, suicide attempts and suicide death in depressive disorder: a Systematic review and meta-analysis. European Archives of Psychiatry And Clinical Neuroscience, 274 (1), 1543–1563. https://doi.org/10.1007/s00406-023-01716-5 (Riera-Serra et al., 2024)	Systematic review	19 studies	• Severe depression • Psychotic features • Guilt Sleep disturbance
Overholser, J. C., Braden, A., & Dieter, L. (2012). Understanding suicide risk: identification of high-risk groups during high-risk times. Journal of clinical psychology, 68 (3), 349–361. https://doi.org/10.1002/jclp.20859 (Overholser et al., 2012)	Evaluation & theroetical model	148 suicides	• Stressful life events • Depressive disorder Prior suicide attempt
Yoshimasu, K., Kiyohara, C., Miyashita, K. et al. Suicidal risk factors and completed suicide: Meta-analyses based on psychological autopsy studies. Environ health Prev Med 13, 243–256 (2008). https://doi.org/10.1007/s12199-008-0037-x	Systematic review	24 articles	• Substance abuse • Mood disorders • Deliberate self-harm Family issues little effect
Favril, L., Yu, R., Uyar, A., Sharpe, M., & Fazel, S. (2022). Risk factors for suicide in adults: Systematic review and meta-analysis of psychological autopsy studies. BMJ Mental Health, 25 (4), 148–155. https://doi.org/10.1136/ebmental-2022-300549 (Favril et al., 2022)	Systematic review	37 psychological autopsy studies	• Clinical factors • History of self-harm Life events smaller
Portzky, G., Audenaert, K. & van Heeringen, K. Suicide among adolescents. Soc Psychiat Epidemiol 40, 922–930 (2005). https://doi.org/10.1007/s00127-005-0977-x	Interviews	32 informants	• Multiple negative life events • Difficulty making friends Depressive episodes
Chen, E. Y. H., Chan, W. S. C., Wong, P. W. C., Chan, S. S. M., Chan, C. L. W., Law, Y. W., … YIP, P. S. F. (2006). Suicide in Hong Kong: a case-control psychological autopsy study. Psychological Medicine, 36 (6), 815–825. https://doi.org/10.1017/S0033291706007240 (Chen et al., 2006)	Case-control psychological autopsy method	150 suicides	• Unemployed • Living alone • Debt • Relationship issues Psychiatric factors
Appleby, L., Cooper, J., Amos, T., & Faragher, B. (1999). Psychological autopsy study of suicides by people aged under 35. British Journal of Psychiatry, 175 (2), 168–174. https://doi.org/10.1192/bjp.175.2.168 (Appleby et al., 1999)	Psychological autopsy study with case–control design	84 studies	• Single • Psychiatric treatment • Psychotropic medication • Life events in last 6 months • Life events in last wee Relationship issues
